# A Robust Method of Measuring Other-Race and Other-Ethnicity Effects: The Cambridge Face Memory Test Format

**DOI:** 10.1371/journal.pone.0047956

**Published:** 2012-10-30

**Authors:** Elinor McKone, Sacha Stokes, Jia Liu, Sarah Cohan, Chiara Fiorentini, Madeleine Pidcock, Galit Yovel, Mary Broughton, Michel Pelleg

**Affiliations:** 1 Research School of Psychology, Australian National University, Canberra, ACT, Australia; 2 Australian Research Council Centre of Excellence in Cognition and Its Disorders, Australian National University, Canberra, ACT, Australia; 3 State Key Laboratory of Cognitive Neuroscience and Learning, Beijing Normal University, Beijing, People's Republic of China; 4 Vision Sciences Laboratory Department of Psychology, Harvard University, Cambridge, Massachusetts, United States of America; 5 School of Psychological Sciences and the Sagol School of Neuroscience, Tel Aviv University, Tel Aviv, Israel; Royal Holloway, University of London, United Kingdom

## Abstract

Other-race and other-ethnicity effects on face memory have remained a topic of consistent research interest over several decades, across fields including face perception, social psychology, and forensic psychology (eyewitness testimony). Here we demonstrate that the Cambridge Face Memory Test format provides a robust method for measuring these effects. Testing the Cambridge Face Memory Test original version (CFMT-original; European-ancestry faces from Boston USA) and a new Cambridge Face Memory Test Chinese (CFMT-Chinese), with European and Asian observers, we report a race-of-face by race-of-observer interaction that was highly significant despite modest sample size and despite observers who had quite high exposure to the other race. We attribute this to high statistical power arising from the very high internal reliability of the tasks. This power also allows us to demonstrate a much smaller within-race other ethnicity effect, based on differences in European physiognomy between Boston faces/observers and Australian faces/observers (using the CFMT-Australian).

## Introduction

In this article, we use the term *race* of a face to refer to the relatively large physical differences in faces with ancestry from different major world regions, such as Europe, Asia, or Africa. We use the term *ethnicity* of a face to refer to the smaller physical differences that exist within a race, such as with ancestry from Norway versus Greece within Europe, or China versus Japan within Asia, or Nigeria versus Ethiopia within Africa.

Previous studies have assessed the effects on memory of both these types of variation in faces. The *other-race effect*, also known as the *own-race bias* or *other-race deficit*, is well established (e.g., [1], for review). Here, individuals of another race are remembered more poorly that those of one's own race, as demonstrated in a two-way interaction between race of observer (e.g., Asian, European), and race of face (Asian, European). [Note: In the face recognition literature, the more common term for “European” is “Caucasian”; however, use of this term here is precluded by PLoS ONE terminology rules.] This interaction is important because it demonstrates that the good memory for own-race faces, and poor memory for other-race faces, is a genuine effect of the match-versus-mismatch in race between observer and face stimulus, and not merely due to one stimulus set containing an easier set of faces (e.g., faces that are more physically different from each other within the set). There is also moderately strong evidence suggestive of an *other-ethnicity effect* on memory [2]–[5], with, for example, Black South Africans showing better memory for Black South African faces than for African-American faces, or German Europeans showing better memory for German than Turkish faces. Importantly, however, only one of these ethnicity studies tested the full crossover design (both observer groups crossed with both types of faces) and reported a significant two-way interaction (in Turkish/German participants crossed with Turkish/German faces [4]).

Traditionally, other-race and other-ethnicity effects have been measured using simple old-new recognition tasks to assess memory — for example, learn 15 faces and later discriminate these within a 30-item test list (half old, half new). In other areas of face recognition research, however, there has recently been strong interest in using the Cambridge Face Memory Test (CFMT) format. This test [6] was initially developed for use in neuropsychological studies of impaired face memory. Since its introduction in 2006, it has become widely used in single-case neuropsychology (e.g., [2], [7]–[12]), in group studies of clinical disorders (e.g., autism spectrum disorder [13]), in individual differences studies of the predictors of face recognition ability (e.g., [14], [15]), and in twin studies of heritability [16].

The CFMT format offers several strengths. First, theoretically, there is good evidence that it provides a valid test of *face* memory, rather than merely memory for a particular photograph, or an individual's general memory ability. As evidence, the stimuli include view and lighting changes to assess face rather than picture memory; hair and clothing are excluded; the test shows only modest correlation with other visual memory (abstract art [16], cars [17]) and no correlation with verbal memory [2]; and the test provides a reliable method of diagnosing clinical impairment in face recognition ability [6]. Second, from a practical perspective: it produces a wide range of scores in the normal population (i.e., there is no problem with ceiling effects); the administration time is relatively quick (10–15 mins per participant); and the test has very high internal reliability. This latter point may be of particular importance. Internal reliability is typically not reported in face recognition studies, but some relevant data comes from Zhu et al [18]: for an old-new recognition list containing 20 faces at study and 40 at test, split half reliability was only .53. This means that each participant's score contains a substantial component of measurement error, which will lower p-values in significance tests. In contrast, the internal reliability of the CFMT is typically .86–.90 [2] [16], which should give it good power for detecting other-race and other-ethnicity effects.

Given these strengths, we expect that many researchers will want to use the CFMT format for the study of other-group effects. Here, our aim is to facilitate such research by (a) introducing a Chinese face version of the task, and (b) demonstrating that the CFMT format provides a robust method of tapping the other-race effect, and indeed is also able to pick up a more subtle other-ethnicity effect within a race.

## Experiment 1: Other-race effect

Experiment 1 tested European (i.e., “White” or “Caucasian”) Australians and Asian participants on two versions of the CFMT: the original, which displays European faces [6], and a newly-created version identical in format that displays Chinese faces (CFMT-Chinese). All participants were tested in Australia: the Asian participants were overseas students.

### Methods

*European* participants were defined as having all known ancestors of European origin (this included British) whose face exposure history was primarily to European faces: that is, the participants were raised in Australia or other majority-European country (e.g. New Zealand, UK, USA, Germany, Poland). *Asian* participants were defined as all having known ancestors of East or South-East Asian origin whose face exposure history was primarily to Asian faces: they were international students from East Asian and South-East Asian countries (e.g. mainland China, Hong Kong, Malaysia, Singapore, Indonesia), and were primarily of Chinese ethnic heritage. Participants were 20 Europeans (9 male, 11 female; age range 18–33 years, *M* = 21.7, *SD* = 4.3) and 24 Asians (9 male, 15 female; age range 19–22 years, *M* = 20.3, *SD* = 1.3). Each observer completed each CFMT version (original, Chinese), with task order counterbalanced across participants.

Participants received $6 for the 30 min study or the option of course credit. The study was approved by the Human Research Ethics Committee of the Australian National University. Participants provided written informed consent.

The CFMT-original was presented using the standard procedure (for details, see [6]). Briefly, participants learn 6 different male faces, each in three views. During the initial “Learn” phase, participants must discriminate the just-learned face from two distractors, with the target face shown in the same image as learned (18 trials, i.e., 6 faces×3 views). In the subsequent “Novel Images” stage, participants discriminate a learned target (which can now be any of the 6 individuals) from two distractors, with the target shown in different viewpoint and/or lighting from the learned photograph (30 trials). The final “Novel Images with Noise” stage uses the same format, with a new set of viewpoint/lighting conditions, and visual noise added to the stimuli to increase task difficulty (24 trials). All faces are shown without hair or clothing. Face stimuli are European, photographed at Harvard University. Regarding internal reliability, Cronbach's alpha (split half reliability taking into account all possible splits) has previously been reported to be .88 [2] and .86–.90 [16], in European or majority-European populations.

**Figure 1:**
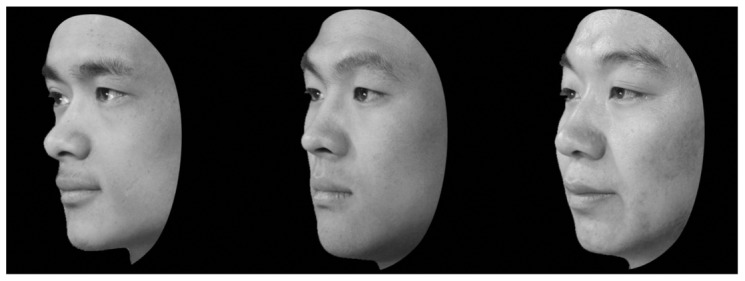
Illustrative appearance of face stimuli in the CFMT-Chinese. Format of face stimuli in the CFMT-Chinese matches that used in previous CFMT tests (CFMT-original and CFMT-Australian). Appearance is illustrated here using individuals not shown in the actual tests, but other individuals from the same population photographed in the same manner.

Our newly-created CFMT-Chinese followed exactly the same procedure, and used face stimuli developed in the same way (i.e., all males, same hair cut-outs, same viewpoint and lighting variation, same noise level; see Figure 1). The Chinese faces were photographs of graduate students (all Han Chinese) at the Chinese Academy of Sciences in Beijing, with written consent forms being collected before photographing. Pilot testing on Chinese observers at Beijing Normal University was used to adjust difficulty level via selection of target and distractor items. Copies of the CFMT-Chinese test are available from Jia Liu of Beijing Normal University (contact: liujia@bnu.edu.cn).

On both tasks, scores are reported as percent correct across the full test. Chance score is 33%. (See Table 1 for CFMT-Chinese scores for each individual stage; these replicate the pattern usual with CFMT-style tests that the Learn stage is at ceiling for own-race observers, as desired, with the other stages more difficult.)

**Table 1:** CFMT-format tasks accuracy (% correct) in both Experiments.^a^

Exp	Observers	Test version	Stage	N	Mean	SD
1	European	CFMT-original	Total	20	75.97	11.69
1	European	CFMT-Chinese	Total	20	65.97	14.43
1	Asian	CFMT-original	Total	24	72.97	15.88
1	Asian	CFMT-Chinese	Total	24	84.61	11.75
			Learn	24	98.48	3.06
			Novel	24	79.24	17.06
			Noise	24	80.11	16.31
2	Harvard	CFMT-original	Total	31	75.54	13.12
2	Harvard	CFMT-Australian	Total	31	74.55	13.09
2	Australian	CFMT-original	Total	68	75.64	13.07
2	Australian	CFMT-Australian	Total	68	79.49	11.08

a This table is provided in addition to the plots so that standard deviation for individual conditions can be reported (the SD is not derivable from the difference-score error bar reported in Figure 2). This could be of value to researchers developing norms for the different tests on different samples for clinical use (prosopagnosia diagnosis), particularly for the new CFMT-Chinese test (note large-N norms for the other versions have already been reported, e.g., see [25]). Also, because the CFMT-Chinese is new, we present results for all three stages separately, to confirm that it shares with the established versions the property that the Learn stage is at ceiling in own-race observers, while the other stages are more difficult. Total = scores for full test; Novel = Novel Images stage; Noise = Novel Images with Noise stage.

### Results and Discussion

Figure 2a (also Table 1) shows mean CFMT-original and CFMT-Chinese scores for European and Asian participants. A two-way ANOVA revealed no main effect of Test Version, F(1,42) = 0.261, MSE = .006, p>.6, indicating that the new CFMT-Chinese is well matched in overall difficulty to the CFMT-original. There was a just-significant effect of participant race, with Asians performing better overall than Europeans, F(1,42) = 4.246, MSE = .031, p = .046; the reason for this is unclear, but one speculation is that individuals willing and able to live in another country to study differ from local students on some variable relevant to face recognition (e.g., higher extraversion, which is associated with better face recognition [19]).

**Figure 2:**
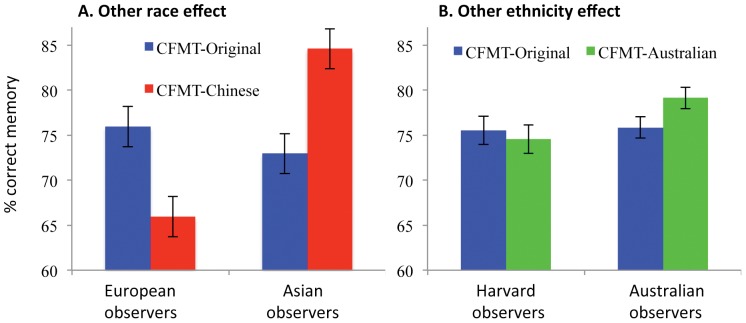
Results. **A.** Results of Experiment 1, showing memory accuracy (% correct) as a function of race of face (CFMT-original = European; CFMT-Chinese = Asian) and race of observer. **B.** Results of Experiment 2: all faces and observers are European, and results show memory accuracy as a function of within-European ethnicity (CFMT-Original = Harvard/Boston faces; CFMT-Australian = Australian faces). In both plots, the presence of an other-race/other ethnicity effect is indicated when the outside bars (match conditions) are higher than the inside bars (mismatch conditions). Error bars show ±1 SE of the difference scores between the two tests, i.e., the appropriate error bar for the within-subjects comparison of the two test versions.

Most importantly, there was also a highly significant other race effect, that is, an interaction between race-of-face and race-of-observer, F(1, 42) = 45.86, MSE = .006, *p*<10^−7^ (specifically, p = .00000003), that arose from poorer memory for other-race faces than for own-race faces (i.e., outer bars higher than inner bars in Figure 2a). Follow-up t-tests found the Asian sample performed significantly better on the CFMT-Chinese than on the CFMT-original, *t*(23) = 5.21, *p*<.001. And, the European sample performed significantly better on the CFMT-original than on the CFMT-Chinese, *t*(19) = 4.44, *p*<.001.

Given that the CFMT-Chinese is a new test, its internal reliability is also relevant. Cronbach's alpha for the new test was .90 in our Asian sample (i.e., same-race observers), and .89 in our European observers. Thus the very high internal reliability of the original version extends to the new Chinese version. For the CFMT-original results were consistent, with alpha of .86 for the European observers and .94 for the Asian observers.

Taken together, these results argue that the CFMT format provides high statistical power in tapping the other-race effect, as would be predicted from the high internal reliability of the tasks. This interpretation is supported by comparison to results from a previous old-new recognition study testing the same population of Europeans as in the present paper. In that previous study [20], European observers drawn from the same Australian National University population were tested on an old-new memory task (for each race-of-face, learn 16 faces, test 32 faces, shown in the same image at study and test; intrinsic difficulty of the Asian and European stimulus sets was matched via distinctiveness ratings from own-race observers). The memory advantage for European faces was 11% (on Hits – False Alarms measure), and with N = 25 this difference was only modestly significant (p = .021). In contrast, the present study found European observers showed a slightly smaller memory advantage for European faces of 10% (i.e., CFMT-original *minus* CFMT-Chinese) and yet with fewer participants (N = 20), this difference was significant at p<.001. That is, a smaller difference between the means has come out to be *more* significant — and, given that the sample size was also smaller, to have a larger statistical “effect size” in terms of percentage-of-variance explained — because the variance for the analysis has been reduced by using a memory task with higher internal reliability. Also note that this enhanced power did not come at the cost of longer testing duration: in fact, the CFMT format required *shorter* testing time (30 min session here, compared to 45 min session in the previous study).

## Experiment 2: Other-ethnicity effect within Europeans

Theoretically, other-ethnicity effects within a race are likely to be smaller than other-race effects, either because the physical differences between the faces are smaller (a face perception explanation, cf. [21]), and/or because social out-grouping may occur less strongly or for fewer of the stimulus faces (a social psychology explanation, cf. [22], [23]). Even using Germanic versus Turkish faces – which on average differ both physically and socially more than do most ethnic groups within Europe – only one of two attempts has revealed a significant within-race ethnicity-of-face by ethnicity-of-observer interaction [4] and this effect seems to have been at best very weakly replicated in [24] (see their Figure 1). Here, we demonstrate that the CFMT format can reveal a significant other-ethnicity effect even for a rather subtle difference in facial appearance between Europeans.

**Figure 3:**
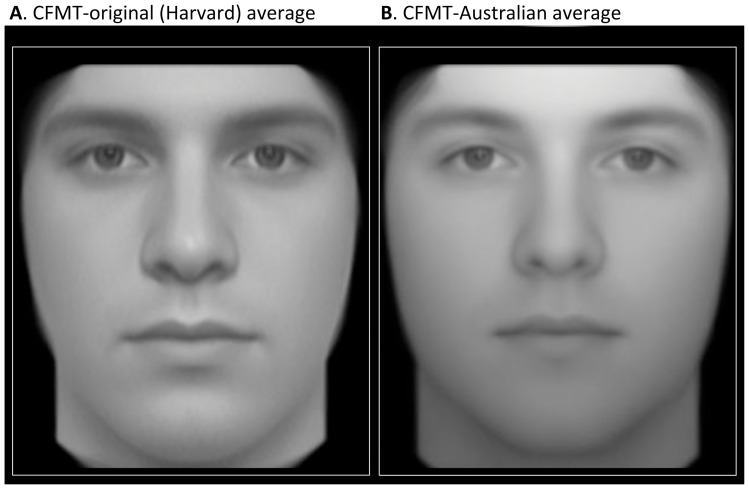
Average faces. **A.** Average face created via morphing procedures from the individuals included in the CFMT-original (Harvard/Boston). **B.** Average face created via morphing procedures from the individuals included in the CFMT-Australian (Canberra, Australia). The image sizes are matched for distance between eyes. The white boxes are identical to facilitate comparison of face width. Note the many differences in both local features and aspects of global face structure. Figure adapted from [25], where method of creating the averages is described.

The CFMT-original faces were photographed at Harvard University, drawing on Harvard students and the Boston community. We contrasted this test with the CFMT-Australian, for which faces were photographed in Canberra at the Australian National University. The Harvard/Boston and Canberra populations differ in demographics. Previously [2], we estimated the proportion of various demographic groups to be: Jewish 35% (Harvard) versus less than 0.5% (Canberra, Australia); Italian 11.8% (Boston) versus 2.6% (Canberra); and, in contrast, British 71% (Canberra) versus 33% (Boston). Correspondingly, there are physiognomic differences between the “average face” created by morphing together the CFMT-original face stimuli, and the average face for the CFMT-Australian (see Figure 3). These can be described as the Harvard average being somewhat more Southern European or Mediterranean in appearance and the Australian average more British or Northern European in appearance. (Note that this does not, of course, mean that *all* faces in the stimulus sets differ in ethnicity — there are some CFMT-original faces that appear British, and some CFMT-Australian faces that appear Southern European — but it indicates that, on average there are ethnicity differences between the sets).

To look for an other-ethnicity effect, we then tested participants from Harvard University, and participants from the Australian National University. All participants were European. Note that no mention was made to participants of different groups, or different ethnicities, or different origins of face stimuli; participants were simply told that “now you are going to do another face learning test”. Thus, any social categorisation processes that participants might have engaged in must be driven by bottom-up information from the facial appearances, not top-down information about group membership provided by the experimenter.

### Methods

For the primary experiment, there were 31 participants from Harvard University (10 male, 21 female; age range 18–23 years, M = 19.5, SD = 1.4), and 68 from the Australian National University (25 male, 43 female; age range 18–23 years, M = 20.3, SD = 1.4; all raised in Australia or New Zealand; two-thirds reported ancestry 100% from the British Isles). Participants received $6 for the half-hour of testing, or course credit. Each participant was tested on the CFMT-original, and the CFMT-Australian. The CFMT-Australian is identical to the original except for use of Australian faces. Its Cronbach's alpha = .88 (for full description of test, see [25]).

The Harvard participants were tested on the tasks in counterbalanced order. For the Australian observers, we tested some participants using counterbalanced order (n = 19) and, given the likelihood that the other-ethnicity effect would be smaller than the other-race effect, we wished to boost power for detecting any effect by including the remaining participants (n = 49), who had all completed the CFMT-Australian first and the CFMT-original second (on the next day; this is a subset of participants reported in [25], matched to the Harvard participants for age). For this inclusion to be valid, we first had to demonstrate that test scores are unaffected by order. This has already been demonstrated with large sample sizes for *CFMT-original*, where scores are unaffected by either practice or interference effects from previous face learning (mean for CFMT-original run first = 76.5% n = 114; mean for CFMT-original after CFMT-Australian = 76.1% n = 75 [25]). To assess possible order effects for the *CFMT-Australian*, we used data from a total of 64 participants who had been tested in counterbalanced order: the 31 Harvard and 19 Australian participants from the main experiment, plus 20 extra participants from Israel (University of Tel Aviv undergraduates, 10 female, age M = 22.45 yrs SD = 1.6; note Israeli subjects are not perfectly matched in ethnic exposure to either of the face stimulus sets, so we do not report their results regarding the main analysis of the other-ethnicity effect). We compared CFMT-Australian scores for participants who completed the CFMT-Australian first, and for those who completed the CFMT-Australian second, following the CFMT-original. There were no order effects. A two-way ANOVA found no suggestion of any main effect of test position (first, second), F(1,64) = 0.493, MSE = 139.995, p>.48, and no interaction between test position and participant group, F(2,64) = 0.559, MSE = 139.995, p>.57 (Australian participants CFMT-Australian done first = 77.8% correct, CFMT-Australian done second = 77.8; Israeli participants first = 80.1, second = 80.1; Harvard participants first = 78.5; second = 72.4). Thus, we combined the “counterbalanced” and “non-counterbalanced” subsets of Australian participants together.

The study was approved by the Human Research Ethics Committee of the Australian National University, the Tel Aviv University ethics committee, and Harvard University's Committee on the Use of Human Subjects in Research. Participants provided written informed consent.

### Results and Discussion

Figure 2b shows mean CFMT-original and CFMT-Australian scores for Harvard and Australian participants. A two-way ANOVA revealed no main effect of participant group, F(1,97) = 0.960, MSE = 267.251, p>.3.

There was no main effect of Test Version, F(1,97) = 1.258, MSE = 45.144, *p* = .26. This provides important evidence that the CFMT-Australian is well matched in overall difficulty to the CFMT-original. Previously, McKone, Hall et al [25] reported that the mean CFMT-Australian in Australian participants matched Duchaine and Nakayama's [6] mean for the CFMT-original in Harvard participants. However, this is the first study to test both versions in both ethnicities of participant.

Turning to the other-ethnicity effect, the 2-way ANOVA detected the presence of a significant interaction between test version (CFMT-Original versus CFMT-Australian) and origin of observer (i.e., Harvard versus Australian), F(1,97) = 4.32, MSE = 45.144, *p* = .04. Follow-up tests showed that, in Australian observers the advantage of CFMT-Australian over CFMT-original was significant, t(67) = 2.76, p<.01, while the reverse trend in Harvard observers was not significant, t(30) = .633, p = .532.

In finding a significant other-ethnicity interaction, note that our sample size, while medium-sized for Harvard participants (n = 31) was larger than might be desired in the future for practical reasons for the Australian participants (n = 68). Thus, we explored whether a smaller sample size could be used and still produce the other-ethnicity effect. Unfortunately, as suggested by the interaction's p = .04 from the entire sample, this did not seem to be the case. We tried reducing the sample size for Australians to n = 31, to match the Harvard sample. We selected a random subsample of 31 out of the 68 Australians, and tested the ethnicity-of-face by ethnicity-of-observer interaction. We repeated this 10 times. Of the 10 random subsamples, all produced a trend in the correct direction (i.e., all still had Australian observers showing CFMT-Australian greater than CFMT-Harvard), but only 4 runs produced a significant interaction (range p = .016 to p = .025), 4 runs produced an interaction approaching significance (range p = .054 to p = .093), and 2 produced very small effects (p = .121, p = .418). Thus, while it might not be necessary to have a sample size quite as large our present one to observe the other-ethnicity effect with our groups, it seems the N could not be reduced by very much.

In summary, the CFMT format was able to reveal a significant other ethnicity effect. The fact that a quite large N was required to achieve this, even with the CFMT format's high internal reliability, does not surprise us theoretically. The difference in average ethnicity between the Harvard faces/observers and the Canberra faces/observers is subtle (e.g., see Figure 3), and probably less than the difference between the Germanic and Turkish faces/observers of Sporer et al [4].

## Discussion

The present article has demonstrated that the Cambridge Face Memory Test format provides an excellent tool for researchers investigating the other-race effect. In Experiment 1, with only a modest number of participants (N = 20 in one race group, N = 24 in the other) the CFMT format produced a very highly significant other-race effect. This was despite the fact that several factors potentially acted *against* finding an other-race effect. First, exposure to the other races was quite high: the Asian observers were not living in Asia but instead had averaged 18 months (SD = 25.7 months) of living in majority-European countries; and the European participants were in a university environment with a large Asian overseas student population. Second, neither race group had a perfect match of the own-*race* faces to their specific *ethnicity*, potentially reducing the own-race scores: that is, not all of the Asian participants were Chinese-heritage as were the Asian faces; and the European participants were mostly Australian-raised but were tested on Boston European faces. Third, in terms of social differences, Asians and Europeans in modern Australia lack the clear differences in status within a social hierarchy that are found in other locations and epochs: that is, Asians are a minority, but are not a low socioeconomic status group.

In Experiment 2, the present study has also been able to demonstrate (using a larger N), that observer's face memory is sensitive not only to large differences between “races”, but also smaller physical differences between “ethnicities” within one race. Face perception researchers have traditionally paid little attention to this idea, with the implicit assumption that any European face set was suitable for any European population anywhere in the world. We have previously shown that using the CFMT-Australian in Australian participants can improve the hit rate for diagnosing developmental prosopagnosia (clinical-level difficulty in recognising faces), compared to using the CFMT-original [25]. The present results demonstrate that, within the nonclinical population, subtle within-race differences in ethnic origin can also affect face memory performance. Our results provide what is, to our knowledge, only the third test of the full crossover design (ethnicity-of-face by ethnicity-of-observer) on face memory, and only the second to find the interaction to be significant (the other being for Turkish/German faces in [4]). It is also worth noting that, while current demonstrations of the other-ethnicity effect have been only in European subpopulations, we presume that the same effect would be present within Asian subpopulations (e.g., anecdotally, individuals raised in Asia say they are able to tell Chinese faces from, say, Korean faces or Japanese faces).

Our final finding concerns the relative size of the other-race and other-ethnicity effects. Comparing across experiments, our results show the other-race effect is substantially larger. We can quantify this by calculating a “face by observer interaction” score from the condition means, as the sum of difference scores, for example: [European observers' European face mean *minus* Asian face mean] *plus* [Asian observers' Asian face mean *minus* European face mean]. This calculation indicates that the other-race effect shifted face memory accuracy by 21.6 points on the %-correct scale, while the other-ethnicity effect within Europeans shifted memory by 4.3 points. Thus, the other-race effect was five times the size of the other-ethnicity effect. (Indeed, the ratio may have been even larger if our first experiment had used perfect match in ethnicity in the own-race condition.) The finding that the other-race effect is larger seems intuitively unsurprising, but it is left for future research to determine whether this arises because (a) the greater physiognomic differences in the face stimuli for race than ethnicity produce greater differences in perceptual coding (e.g., in degree of appropriateness of dimensions in face-space coding [21]; or in greater reduction in holistic processing, c.f. [26]), and/or (b) for social psychological reasons (e.g., because participants categorise more of the other-race than other-ethnicity faces as out-group members, c.f. [22]).

In conclusion, the present article has demonstrated that the CFMT format showed a robust other-race effect, and was also able to reveal a smaller effect of subtle differences in ethnicity within a race. Together with the demonstrated theoretical validity of the CFMT as a test of face memory rather than general memory (see Introduction), this confirms the suitability of the CFMT format for investigations of other-race and other-ethnicity effects. Note that the format's high internal reliability leads not only to good power for studies using group-level analyses (i.e., comparing means) of these effects, but also makes the tasks suitable for correlational studies of other-race and other-ethnicity effects utilising individual differences between participants. Finally, the high reliability of our new CFMT-Chinese also makes it suitable for individual-case analysis (e.g., for diagnosis of prosopagnosia in Chinese individuals).
